# Deep sequencing of HPV16 genomes: A new high-throughput tool for exploring the carcinogenicity and natural history of HPV16 infection

**DOI:** 10.1016/j.pvr.2015.05.004

**Published:** 2015-06-15

**Authors:** Michael Cullen, Joseph F. Boland, Mark Schiffman, Xijun Zhang, Nicolas Wentzensen, Qi Yang, Zigui Chen, Kai Yu, Jason Mitchell, David Roberson, Sara Bass, Laurie Burdette, Moara Machado, Sarangan Ravichandran, Brian Luke, Mitchell J. Machiela, Mark Andersen, Matt Osentoski, Michael Laptewicz, Sholom Wacholder, Ashlie Feldman, Tina Raine-Bennett, Thomas Lorey, Philip E. Castle, Meredith Yeager, Robert D. Burk, Lisa Mirabello

**Affiliations:** a Division of Cancer Epidemiology and Genetics, National Cancer Institute, National Institutes of Health, Rockville, MD, USA; b Cancer Genomics Research Laboratory, Frederick National Laboratory for Cancer Research, Leidos Biomedical Research, Inc., Frederick, MD, USA; c Department de Biologia Geral, Instituto de Ciências Biológicas, Universidade Federal de Minas Gerais, Belo Horizonte, Brazil; d Frederick National Laboratory for Cancer Research, Simulation, Analysis and Mathematical Modeling Group Advanced Biomedical Computing Center, Frederick, MD, USA; e Thermo Fisher Scientific (Formerly Life Technologies), Carlsbad, CA, USA; f Department of Epidemiology and Population Health, At Albert Einstein College of Medicine, Bronx, NY, USA; g Regional Laboratory and Women’s Health Research Institute, Division of Research, Kaiser Permanente Northern California, Oakland, CA, USA; h Global Coalition Against Cervical Cancer, Arlington, VA, USA; i Division of Gynecologic Oncology, Department of Obstetrics & Gynecology and Women’s Health, at Albert Einstein College of Medicine, Bronx, NY, USA

**Keywords:** HPV16, HPV epidemiology, HPV genomics

## Abstract

For unknown reasons, there is huge variability in risk conferred by different HPV types and, remarkably, strong differences even between closely related variant lineages within each type. HPV16 is a uniquely powerful carcinogenic type, causing approximately half of cervical cancer and most other HPV-related cancers. To permit the large-scale study of HPV genome variability and precancer/cancer, starting with HPV16 and cervical cancer, we developed a high-throughput next-generation sequencing (NGS) whole-genome method. We designed a custom HPV16 AmpliSeq™ panel that generated 47 overlapping amplicons covering 99% of the genome sequenced on the Ion Torrent Proton platform. After validating with Sanger, the current “gold standard” of sequencing, in 89 specimens with concordance of 99.9%, we used our NGS method and custom annotation pipeline to sequence 796 HPV16-positive exfoliated cervical cell specimens. The median completion rate per sample was 98.0%.

Our method enabled us to discover novel SNPs, large contiguous deletions suggestive of viral integration (OR of 27.3, 95% CI 3.3–222, *P*=0.002), and the sensitive detection of variant lineage coinfections. This method represents an innovative high-throughput, ultra-deep coverage technique for HPV genomic sequencing, which, in turn, enables the investigation of the role of genetic variation in HPV epidemiology and carcinogenesis.

## 1 Introduction

The human papillomaviruses (HPVs) of the genus *Alphapapillomavirus* are widely prevalent among human populations, infecting anogenital and oral mucosal and cutaneous epithelia [1]. Although most HPV infections are benign, specific types of HPV infections have been estimated to cause approximately 610,000 cancers worldwide each year [2]. In fact, persistent infection with one of a dozen carcinogenic HPV types is a well-established, necessary cause of cervical cancer [3], the third leading cause of cancer in women worldwide [4]. HPV also causes a large proportion of vaginal, vulvar, penile, anal, and oropharyngeal cancers. Despite the pervasiveness of “high-risk” HPV (HR-HPV) infections, only a small fraction of women with a HR-HPV infection at any infected site will progress to precancer or cancer [5], [6]. This indicates that additional risk factors are important for HR-HPV carcinogenesis, potentially including viral genetic factors; however, these factors remain poorly understood.

HPVs are small and evolve slowly, at nearly the same rate as the human genome, since they use the host replication machinery. HPV’s have a circular double-stranded DNA genome of approximately 8000 bases, encoding eight viral proteins on one strand using all three coding frames [3]. The HPV genome consists of an early region (E1, 2, 4–7 genes), which codes for core viral functions, including the E6 and E7 ORF, which are linked to cellular transformation. The late (L1-2 genes) region codes for the viral capsid proteins. A non-coding upstream regulatory region (URR) contains DNA-protein binding motifs and is involved in replication. Each unique HPV type is defined as differing from all other characterized HPV types by at least 10% in the highly-conserved L1 nucleotide sequence [7]. We know that viral genetic factors are important because, as mentioned above, HPV types differ profoundly in carcinogenicity [8]. HPV type 16 (HPV16) is the most important and potent carcinogenic HPV type, identified in approximately 50% of cervical precancers and up to 60% of cervical cancers [8], [9], and the large majority of other HPV-induced malignancies.

Cervical cancer is the most important and best understood site of HPV carcinogenesis. In terms of absolute risk, persistent cervical infection with HPV16 is among the most potent human carcinogens [10]. By contrast, genetically closely related HPV31 and HPV35 are much less common and much less carcinogenic than HPV16, each causing less than 5% of cervical cancers [8]. Therefore, important genetic information linked to carcinogenic potential must be embedded in the small HPV genome [11].

Strikingly, there is large variability in precancer/cancer (CIN3+) risk within HPV16 by variant lineage [12]. The nucleotide sequences of HPV variant lineages differ from each other by 1% to less than 10% across the entire genome [12]. HPV16 can be classified into 4 main variant lineages related to human population origins and movement. Although variants of HPV16 differ by only approximately 200 nucleotides, the non-European HPV16 variant lineages are associated with an increased risk, estimated up to 10-fold, of invasive cancer compared to European variants [13], [14], [15], [16], [17], [18], [19], [20], [21], [22].

Given the large differences in cancer risk associated with limited definable genotypic variation, it should be possible to determine specific SNPs or “viral haplotypes” (i.e., fixed variations across the viral genome) responsible for increased viral carcinogenicity. The laboratory and analytic approach can be extended to HPV16-related types, and eventually all HR-HPV types. However, even with the relatively simple model of HPV16 genomic variability, in order to have statistical power to interrogate thoroughly the genetic basis of HPV16 carcinogenicity, complete HPV genome sequencing of many thousands of viruses from large population-based studies of benign HPV16 infections and precancer/cancer are needed [12]. The labor, time and budget to sequence these large-scale HPV studies with current Sanger sequencing methods are prohibitive. With the recent advent of next-generation sequencing (NGS), determining the genetic basis for this variability is now achievable.

Specifically, in response to the need for a high-throughput, cost-effective, and robust HPV sequencing process, we have developed a novel NGS assay, combining Ion AmpliSeq library construction and Ion Torrent Sequencing technology [23]. Here, we describe the novel method that permits the study of genetic variability within HPV16 in relation to cervical cancer risk. We whole-genome sequenced 796 HPV16-positive exfoliated cervical cell specimens from the Kaiser Permanente Northern California NCI HPV Persistence and Progression (PaP) cohort [24] and give examples of novel insights enabled by this new method to the study of HPV epidemiology.

## 2 Materials and methods

### 2.1 Study population

The HPV PaP cohort is a repository of residual cervical specimens from approximately 55,000 women obtained during routine cervical cancer screening and testing at Kaiser Permanente Northern California (KPNC). By design, the study focus is to determine risk factors for precancer and cancer among HPV-positive women; thus, 45,000 of the specimens in PaP were selected because they were HPV-positive at baseline. These baseline specimens were collected in specimen transport medium (STM; Qiagen Inc., Gaithersburg, MD, USA) between January 2007 and January 2011 [24].

In KPNC, women 30 and older were routinely screened every 3 years for cervical cancer by “cotesting”, i.e., by testing for a pool of 13 carcinogenic HPV using the Hybrid Capture 2 (HC2; Qiagen) test in addition to obtaining cervical cytology. Also, women aged less than 30 with an equivocal Pap smear were triaged using the HC2 test. Eligibility was defined as specimens from women 21 and older who had not opted out from having their specimen banked and tested for HPV-related biomarkers including HPV genotypes. De-identified data including age and follow-up cytologic and pathologic results were obtained from electronic health records.

Our study included baseline exfoliated cervical cell specimens from 796 HC2-positive women, previously found by a research-use-only typing test to contain HPV16 DNA [25]: the study population included 472 CIN3 precancer cases and 69 cancer cases, and 255 controls. The cases were diagnosed at enrollment; in addition, a few original controls developed CIN3 during the 5-year study follow-up period (*n* = 11) and were reclassified as cases. The controls were defined as baseline specimens with HPV16 DNA and no cytologic or histologic evidence of even equivocal precancer (CIN2+) during the follow-up study period.

### 2.2 DNA isolation, HPV16 detection, and viral load estimation

HC2 was conducted on STM specimens as part of routine cervical cancer screening at KPNC enrollment per the manufacturer’s instructions. DNA was extracted from the banked STM specimens as previously described [26]. We have used a simple DNA isolation technique: 100 ul of STM was incubated with a solution containing Laureth12 and Proteinase K, and thereafter precipitated with an ammonium acetate/ethanol precipitation solution and resuspended in 100 ul of a TE solution. The MY09/M11 L1 degenerate primer PCR (MY09/11 PCR) and type-specific dot-blot hybridization methods were used at the Burk laboratory at Albert Einstein College of Medicine, to identify the HPV16-containing HC2 positive STM specimens [26], [27]. Co-infections with other HPV types were ignored. In addition to HPV typing, dot blot oligonucleotide hybridization signal intensity, a measure of viral load, was evaluated by two researchers using a qualitative index on a scale of 1–5 (weakest=1 and strongest=5). The index represents the strength of the hybridization signal established by observing the density and diameter of the PCR product on the autoradiogram. For the current analyses, viral load was collapsed into dichotomous categories of low (PCR signal strength index of 1–3) and high (PCR signal strength index of 4–5) viral load [28].

### 2.3 Ion AmpliSeq library preparation

A custom Ion AmpliSeq HPV16 panel was employed to amplify the entire 7906 bp HPV16 genome as 47 overlapping amplicons ranging in size from 181 bp to 375 bp. Our laboratory designed custom HPV16 degenerate primers using a consensus sequence with ambiguity codes (IUPAC) derived from seven sequences representing major HPV16 lineages (2 European, 1 European/Asian, 1 African-1, 1 African-2 and 2 Asian-American) in order to design degenerate primers for sequencing (Supplemental Table 1). HPV16 reference sequences included: European.prototype, w0122|European.prototype, w0724|European.asian, R872|African-1, R460|African-2, Qv00995|Asian-American, Qv15321|Asian-American. Proprietary nucleotide modifications were made to the degenerate primers which were grouped into two overlapping primer pools.

Libraries were generated following the manufacturer’s Ion AmpliSeq Library Preparation kit 2.0-96LV protocol (Life Technologies, Part #4480441) with modifications highlighted below. In brief, 1 ul of partially purified total DNA from exfoliated cervical cells underwent two separate targeted 10 ul amplification reactions using Ion AmpliSeq HiFi Master Mix and each of two separate non overlapping HPV16-specific primer pools for a total of two amplification reactions per sample. Thirty PCR cycles were performed following the manufacturer’s cycle times and temperatures. Amplicons from both primer pools were combined prior to FuPa digestion and ligation to Ion Xpress Barcode Adapters (1–96) enabling the pooling of 96 samples into one Proton sequencing run. Our initial targeted amplification was so robust that we opted not to perform the optional second amplification step using Ion Torrent specific amplification primers and therefore proceeded to two rounds of Agencourt AMPure XP cleanup using a 0.5x followed by 1.2x bead-to-sample volume ratio to remove input DNA and unincorporated primers from the amplicons. Individual library concentrations were determined using the Roche LightCycler 480 Instrument II (Part #05015278001) using the Kapa Biosystems Library Quantifiication Kit—Ion Torrent/LightCycler 480 (Part #KK4857). Based on qPCR results a total of 6 million templates per sample were pooled. The final pooled library concentration and size distribution was determined using the Agilent 2100 Bioanalyzer (Agilent Technologies Part #G2940CA) with the Agilent BioAnalyzer DNA High-Sensitivity LabChip (Agilent Technologies Part #5067-4626). The pooled library averaged 305 bp in size (size range 266–460 bp).

### 2.4 HPV16 complete genome sequencing: Ion torrent proton & personal genome machine

Template emulsion PCR, emulsion breaking and enrichment were performed using the Ion P1 Template OT2 200 Kit v3 (Part #4488318) according to manufacturer’s instructions. In brief, an input concentration of 0.016 to 0.3 library templates per Ion Sphere Particle (ISP) was combined with emulsion PCR master mix and run on the Ion One Touch 2 (Life Technologies Part #4474779). Enrichment of template-positive ISPs by Dynabead MyOne Streptavidin C1 bead (Life Technologies, Part #65001) capture was confirmed using the EMD Millipore Guava easyCyte (EMD Millipore Part #0500-5008). Sequencing a 96-well plate in its entirety on an Ion Torrent 318 chip (Life Technologies Part #4484355) was carried out using the Ion PGM Sequencing 400 Kit (Life Technologies, Part #4482002) following the manufacturer’s recommended protocols found on the Ion Community Website (ioncommunity.lifetechnologies.com). Sequencing on the Ion PGM Platform requires three sequencing runs to achieve optimal read depth per sample. Sequencing a 96-well plate in its entirety on an Ion P1 Chip Kit v2 (Life Technologies, Part #4482321) was carried out using the Ion PI Sequencing 200 Kit v3.0 (Life Technologies, Part #44885315) following the manufacturer’s recommended protocol (ioncommunity.lifetechnologies.com) and required one sequencing run. Eight water controls and eight HPV16-negative (but human DNA positive) controls were also included (one per plate) to assess potential HPV16 contamination and to determine if there was an influence of human DNA on the specificity of sequencing.

### 2.5 HPV16 complete genome sequencing: Sanger

For a subset of the samples, the complete 8 kb genomes of HPV16 isolates were amplified by overlapping PCR, as previously described [29], [30]. In brief, three sets of primers for nested PCR were designed to amplify the entire genomes in 3 overlapping fragments (Supplemental Table 2). For overlapping PCR, an equal mixture of AmpliTaq Gold DNA polymerase (Applied Biosystems, Carlsbad, CA) and Platinum Taq DNA Polymerase (Invitrogen, Carlsbad, CA) was utilized. PCR products of anticipated size, as determined by gel analyses, were purified and directly sequenced on an ABI 3700 Sequencer in the Einstein Sequencing Facility. Comparison of repeat sequencing of PCR products from the same isolates resulted in a difference of less than one change per 8000 bp. Several additional sequencing primers were used to obtain supplemental sequence to clarify sequence ambiguity and assemble the complete genome.

The nucleotide sequences of HPV16 complete genomes were aligned with each representative lineage and sublineage of HPV16 variants [22] using the program MAFFT v6.864b [31]. A maximum likelihood (ML) tree was constructed using RAxML MPI v7.2.8.27 [32] with default parameters. SNPs within the HPV16 genomes were determined from the global alignment using MEGA5 [33].

### 2.6 Next-generation sequence (NGS) alignment and quality metrics

Because the HPV genome is circular, one of the 47 amplicons was split bioinformatically to create 48 overlapping contigs that were mapped across the genome. Raw sequencing reads generated by the Ion Torrent sequencer were quality and adaptor trimmed by Ion Torrent Suite and then aligned to the HPV16R reference (7906 bp) [34] sequence using TMAP. To filter out human reads, all the read were realigned to hg19-HPV16 hybrid reference genome and only HPV16 reads were kept. Resulting BAM files were merged according to sample names and processed through an in-house quality control (QC) and coverage analysis pipeline, which generated coverage summary plots and per sample per amplicon read count heatmaps. BAM files were then left aligned using the GATK LeftAlignIndels module. Amplicon primers were trimmed from aligned reads.

### 2.7 HPV16 NGS variant calling and annotation database

The HPV16 genome was genotyped by GATK HaplotypeCaller Version 3.3[35]. SNP and indel calls were made and filtered by the Torrent Variant Caller Version 4.2 (http://mendel.iontorrent.com/ion-docs/Torrent-Variant-Caller-Plugin.html). For each sample, a whole-genome sequence fasta file was generated by combining results from TVC and GATK.

A customized HPV16 annotation database was built according to the coordinates of each HPV gene and regulatory regions. All identified HPV16 nucleotide variants were annotated with the following features: HPV16 gene or region, amino acid changes, and transcription factor binding site changes using the SnpEff program [36]. CpG site changes were also annotated with a separate script.

### 2.8 Sequence completion and concordance rates

We evaluated several metrics to assess the quality and reliability of our HPV16 NGS data: overall and per sample genome completion rates, concordance among 18 replicated samples (replicated 1–5 times across each plate, total replicates=28), concordance with Sanger sequencing data, overall HPV16 genome coverage and the coverage distribution. Custom software was used to determine completion rates across the HPV genome, concordance among duplicate samples and concordance between Ion Torrent and Sanger sequencing per sample and per locus. The sequence completion rate was defined as the percentage of the length of the genome sequence and the total number of called positions across the genome, calculated as the percent of the (total sequence positions called)/(genome sequence length). Sequence concordance was defined as the percentage of matching bases at each position of the genome when comparing methods or when comparing duplicates of the same sample, and is used as a method of assessing the accuracy of the assay. Concordance by sample was estimated as the percent of the (total number of nucleotide positions that agree)/(total number of called positions across the genome); and, concordance by position was estimated as the percent of the (total number of sample pairs that agree at a nucleotide position)/(total number of sample pairs with sequence data at that position).

### 2.9 HPV16 variant lineages and co-infection detection

HPV16 variant lineage assignment was based on the maximum likelihood (ML) tree topology constructed using RAxML MPI v7.2.8.27 [32] including 16 HPV16 European and non-European variant lineage reference sequences, and lineage assignments were confirmed with SNP patterns.

An in-house custom program was implemented to identify the heterozygous calls using the VCF sequence files and diagnostic sites. Diagnostic sites were HPV16 nucleotide positions that distinguished HPV16 variant lineages (i.e., positions that were known to be variable between the variant lineages). We built a hash table for the diagnostic sites, and then mapped the diagnostic sites to the associated position in each VCF file. The diagnostic sites that contained more than one genotype call, the heterozygous sites, were identified and compiled in a tab-delimited file with the sample ID, position, depth, genotype and frequency of each identified variant.

**Fig. 1:**
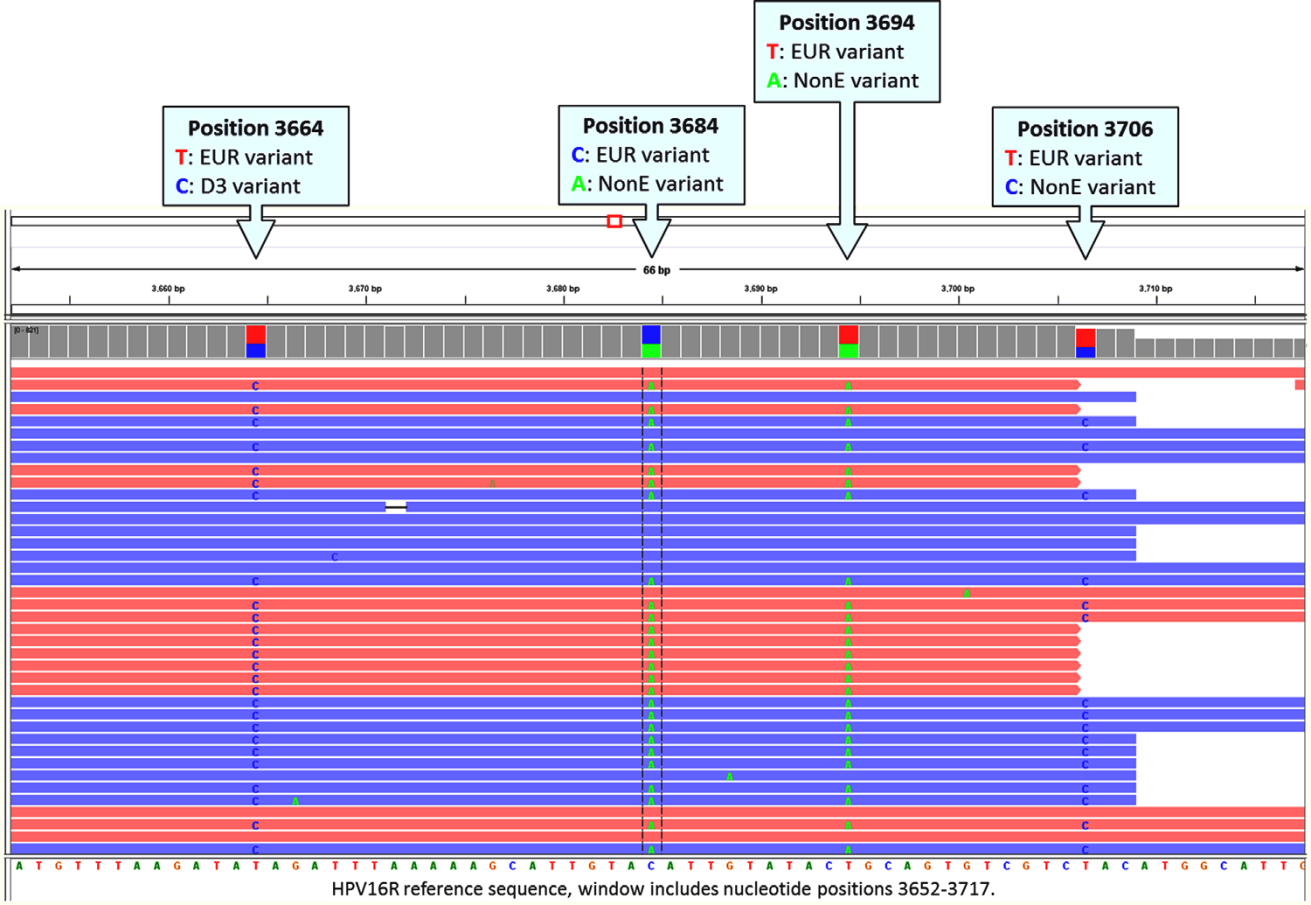
HPV16 variant lineage co-infection identification. Integrative Genomics Viewer (IGV) [37] screenshot showing an example of a European (EUR; A) lineage and non-European (nonE; D3) lineage coinfection. Sequencing reads (forward reads in red and reverse in blue) show consistent variants across four nucleotide sites for both the European and non-European variant lineages. Multiple sites across the HPV16 genome confirm patterns seen in the smaller IGV window. The HPV16 reference nucleotide position is shown first for the variants highlighted in each callout, and the reference sequence is shown along the bottom of the window below the sequence reads. (For interpretation of the references to color in this figure legend, the reader is referred to the web version of this article.)

Suspected co-infections were verified by manually inspecting each woman’s individual sequence reads at each variable nucleotide position to determine if there were more than one HPV16 isolate present. Regions with a high density of variants in close proximity were inspected for the presence of shared SNPs unique to a specific HPV16 variant lineage (e.g., a non-European variant lineage) present in only a proportion of reads, and the other proportion of reads with shared variants unique to a different HPV16 variant lineage (e.g., a European variant lineage) in the same woman. For example, a non-European lineage specific nucleotide change should be present in the same shared reads and a European lineage nucleotide change present in different shared reads in the same woman (see Fig. 1). These lineage specific changes were manually confirmed across the genome.

### 2.10 Identification of HPV16 novel SNPs

For comparative purposes we obtained 62 previously published Sanger whole-genome HPV16 sequences [22]; previously unreported SNPs were classified as “novel”. An in-house custom program was developed to categorize these SNPs. The program used the SNP information from the 62 published sequences [22] as the known SNP baseline. These Sanger SNPs and a SNP list from Ion Torrent, after applying QC to filter out low quality SNPs, were used to build the key and value pair hash tables for both Ion Torrent and Sanger SNPs respectively. The SNPs were then binned into three categories: Ion Torrent only, Sanger only and the overlap between Sanger and Ion Torrent.

Variable sites that were frequently heterozygous were manually inspected with Integrative Genomics Viewer (IGV; www.broadinstitute.org/igv/) [37] and removed from the dataset if poor quality. We only focused on variants that passed the QC filter.

### 2.11 Statistical analyses

The non-parametric Mann–Whitney *U* test was used to determine if HPV16 sequence coverage (expressed as the percentage of amplicons exceeding 25× or 500× sequence coverage) was significantly different between the high and low viral load categories. Differences in the distribution of coverage in the high versus low viral load categories were visualized by a violin plot and confirmed by Kolmogorov–Smirnov testing. A logistic regression model was used to obtain the odds ratio (OR) and 95% confidence intervals (CI) for precancer and cancer using the controls as the referent group. Statistical analyses were performed with SPSS version 21.0 and R version 3.1.2; all statistical tests were two-sided.

## 3 Results

We whole-genome sequenced 796 HPV16-positive specimens and report several metrics to demonstrate the high quality and reliability of our assay and describe HPV16 genome variation based on these NGS data.

### 3.1 HPV16 genome sequencing concordance and completion rates

Sequence concordance was very high among the 18 sample duplicates ranging from 99.7 to 100% (mean 99.97%, standard deviation [SD] 0.07). The median completion rate for 796 specimens was 98.0% (interquartile range 5.6%). To evaluate if the sequence completion rates were related to viral load, we compared the distribution of samples with greater than 25× coverage in the low versus high viral load categories. Viral load was estimated from the signal intensities after dot blot oligonucleotide hybridization and were dichotomized by the Burk laboratory into low (PCR signal strength index of 1–3) and high (PCR signal strength index of 4–5) viral load categories. Sequence completion rates were directly associated with viral load (i.e., samples with a low viral load had fewer reads exceeding 25× coverage; Mann–Whitney *U* test, *P*<0.0001). This relationship is illustrated with a violin plot, which shows the greater variability in coverage for the samples with a lower viral load (Kolmogorov–Smirnov Test; *P*<0.0001) (Supplemental Fig. 1a). The same association held true for samples with coverage exceeding 500× (Supplemental Fig. 1b).

**Fig. 2:**
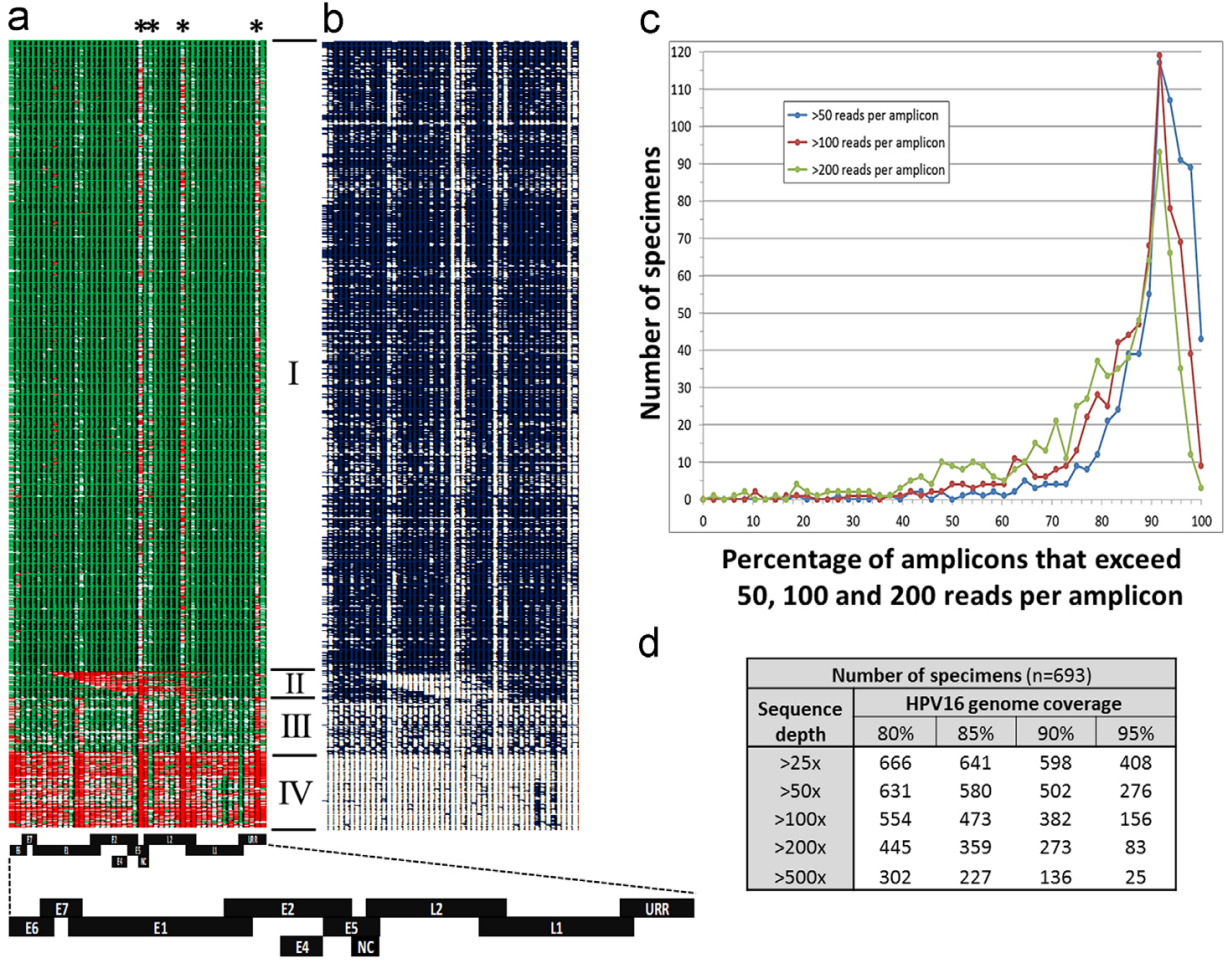
HPV16 sequence depth for 796 samples. (a) Heat map illustrates the sequence depth across 48 overlapping amplicons (columns 1–48) for 796 samples (rows 1–796) displaying sequence depths >100× (green cells), depths of 15× (white cells) and depths <2× (red cells). Asterisks highlight four poorly performing amplicons. High quality sequence for 638 samples far exceeded 25× coverage (group I). Twenty-six samples contained large central deletions (group II). Fifty-five samples yielded high quality and depth sequence data that contained some specific amplicon dropout (group III). Seventy-seven samples performed poorly (group IV). The HPV16 reference map aligns each gene to the corresponding amplicon(s). The exaggerated overlap of adjacent HPV16 genes and regions (early genes: E6, E7, E1, E2, E4, E5; late genes: L2, L1; the upstream regulatory region, URR; non-coding region, NC) in the map reflects the overlapping design of the amplicons. (b) Heat map of sequence depths >500× (blue cells) and less than 500× (white cells). (c) The number of samples (*y*-axis) that exceeded 50× (blue), 100× (red) and 200× (green) sequence coverage (*x*-axis) for one amplicon (2% of genome) up to 48 amplicons (100% of the genome). (d) Summary statistics for the number of high quality group I plus group III samples (n=693) that exceeded 80, 85, 90 and 95 percent sequence coverage at depths of greater than 25×, 50×, 100×, 200× and 500×. (For interpretation of the references to color in this figure legend, the reader is referred to the web version of this article.)

To validate our approach, we determined the concordance between Sanger, representing the current “gold standard” of sequencing, and Ion Torrent sequence data in a random subset of 89 specimens. The mean concordance across the 7906 bp HPV16R genome by sample was 99.97% (SD 0.13; range of 98.9 to 100%) and by genomic position was 99.93% (SD 0.79; range of 55.6 to 100%; Supplemental Fig. 2). There were only two nucleotide positions where the concordance dropped below 85% (concordance of 66.7% and 55.6%; Supplemental Fig. 2); these two outliers were located within the E5 gene in a small region that was a challenge to amplify, illustrated in Fig. 2a.

### 3.2 HPV16 genome coverage and quality

The sequence depth across the 48 overlapping amplicons is illustrated for the 796 total specimens in Fig. 2. For 638 samples (80.1% of samples), the average sequence depth per amplicon was very high (5135×), ranging from 134× to 213,829× coverage (Fig. 2a, group I). Seventy percent of amplicons in this group I were sequenced at depths exceeding 500× (Fig. 2b). Sequence depth per amplicon for 55 samples was high, yet compromised in 24% of the amplicons where coverage fell below 25× (Fig. 2a, group III). Seventy-seven samples sequenced poorly across the entire genome (Fig. 2a, group IV). There were four viral regions of low genomic sequence complexity that were a challenge to amplify due to the need for larger amplicons to bridge these regions corresponding to amplicons 24, 26, 32 and 47 (asterisk, Fig. 2a), located within the HPV16 E5, L2 and URR regions, that accounted for 379 bp or 4.8% of the viral genome. Overall, Ion Torrent sequencing of 96 barcoded samples on a Proton P1 sequencing chip generated high quality sequence data at depths of 25× to over 500× that covered 80 to 100% of the viral genome in a single sequence run (Fig. 2c–d). The water controls and HPV16-negative human DNA positive controls were clear of HPV16 sequence reads with a median sequence depth per amplicon of 1.1×.

### 3.3 Custom HPV annotation pipeline

We designed a custom pipeline for sequence variant calling and annotation. This pipeline allowed us to identify sequence variants and annotate them in a high-throughput manner. All SNPs were annotated for HPV16 genomic position, missense/nonsense/silent change to a coding gene, alteration of a presumed transcription factor binding site (e.g., E2 binding sites [E2BS]), and CpG site change (Table 1).

**Table 1:** Annotated HPV16 genome SNPs in independent specimens detected by NGS.

**Gene/feature**	**Size (bp)**	**Total SNPs**	**% Variable sites**	**“Novel” SNPs**^a^	**No. indels**	**% Novel SNPs**	**Silent**		**Missense**		**Start loss**		**Nonsense**		**% Novel Nonsyn. SNPs**	**CpG site loss**^b^	**CpG site gain**^b^
							**Total**	**Novel**	**Total**	**Novel**	**Total**	**Novel**	**Total**	**Novel**			
**E6**	477	53	11.1	38	1	71.7	15	10	34	25	1	0	3	3	73.7%	0.3%	4.5%
**E7**	297	19	6.4	12	0	63.2	13	7	6	5	0	0	0	0	83.3%	0.2%	0.8%
**E1**	1949	168	8.6	112	1	66.7	96	57	72	55	0	0	1	1	76.7%	1.0%	22.4%
**E2**	1098	122	11.1	77	0	63.1	40	22	84	55	0	0	1	1	65.9%	13.6%	32.4%
**E4**	288	56	19.4	31	3	55.4	28	12	21	17	0	0	2	2	82.6%	8.5%	18.6%
**E5**	252	40	15.9	27	1	67.5	23	15	19	12	0	0	0	0	63.2%	0.0%	4.7%
**L2**	1422	253	17.8	171	1	67.6	115	66	141	105	0	0	1	1	74.6%	1.5%	9.1%
**L1**	1596	156	9.8	121	0	77.6	102	75	58	48	0	0	1	1	83.1%	0.5%	6.5%
**URR**	831	146	17.6	83	3	56.8	–	–	–	–	–	–	–	–	–	5.5%	2.5%
**E2BS⁎**	44	4	9.1	1	0	25.0	–	–	–	–	–	–	–	–	–	–	–

Nonsyn, nonsynonymous; freq, frequency; early genes: E6, E7, E1, E2, E4 (overlaps with the E2 gene region), E5; late genes: L2, L1; upstream regulatory region: URR.

Total number of variable positions, some SNPs had multiple variable alleles that led to multiple changes; ^†^ based on four E2 binding sites (E2BS) in the URR.

a SNPs were considered “novel” if they were not present in the 62 reference HPV16 Sanger sequences [22].

b Frequency of CpG site changes are shown for women with a HPV16 European variant lineage compared to the HPV16 European prototype reference sequence.

For example, our custom annotation pipeline allowed us to automatically track alterations at CpG sites across the viral genome relative to HPV16 variant lineage reference genomes. CpG methylation of the HPV genome is highly associated with precancer as opposed to benign infection [38], [39]; thus, it is conceivable that part of the association of genomic variability and risk of precancer could be mediated by CpG sites. The goal of this analysis was to identify loss of known CpG sites, as well as identify SNPs that created new CpG sites. In comparison to the HPV16 European prototype reference (NC_001526), the HPV16 European variant lineages had the greatest number of known CpG site losses and CpG site gains, particularly in the E2 gene region (Table 1). CpG gains per sample ranged from 0 to 11 and losses ranged from 0 to 4 per sample.

We detected a total of 961 SNPs and 10 indels (insertions or deletions) across the HPV16 genome (summarized in Table 1). Regions with the greatest and least number of SNPs, given their size (i.e., number of SNPs/region size in nucleotides), were L2 and E7, respectively. There were 435 nonsynonymous changes; three resulted in a premature stop (nonsense) within the E2 or E4 gene. We replicated all singleton SNPs occurring in only one sample and all indels for validation, and determined that 84.1% of these singleton SNPs and indels validated. We identified 642 SNPs that were not present in the Smith et al. [22] report of 62 HPV16 genome sequences (termed “novel” SNPs). Fifty-two percent of these novel SNPs led to coding changes. The percentages of non-synonymous SNPs that were novel ranged from 63.2 to 83.3% for each of the eight protein-coding genes.

### 3.4 HPV16 variant lineage risk associations

We determined HPV16 variant lineage assignment based on a phylogenetic maximum likelihood tree for 719 women, excluding the samples with poor read depth (Fig. 2a, group IV). 604 women (84.0%) in our study of women living in Northern California had an HPV16 European variant lineage (A) infection, including the following sublineages: 493 A1, 80 A2, 4 A3, and 27 A4 (Asian). There were 115 women (16.0%) with an HPV16 non-European variant lineage infection: 17 B (African-1), 17 C (African-2), and 81 D (North American/Asian-American). We assessed HPV16 variant lineage associations with cervical precancer and cancer compared to the most common European sublineage, A1 (Table 2). We confirmed that women with an HPV16 non-European lineage (B/C/D) infection had a significantly increased risk of cancer compared to women with an HPV16 European A1 lineage infection (OR of 4.3, 95% CI 2.1–8.5, *P*=4.0×10^−5^; Table 2).

**Table 2:** HPV16 variant lineage risk associations.

**Status**	**Tested variant**	**N**	**Reference variant**	**N**	**OR**	**95% CI**	***P***
Control	Non-EUR, B/C/D	23	EUR, A1	145	1.0		
CIN3		69		314	1.4	0.8–2.3	0.211
Cancer		23		34	4.3	2.1–8.5	4.0×10^−5^

*N*, number of women in each lineage.

OR, odds ratio; 95% CI, 95% confidence intervals.

CIN3, cervical intraepithelial neoplasia grade 3; CIN3+, cervical intraepithelial neoplasia grade 3 and cancer; EUR, European.

### 3.5 Ion AmpliSeq can identify large deletions and HPV16 variant co-infections

**Fig. 3:**
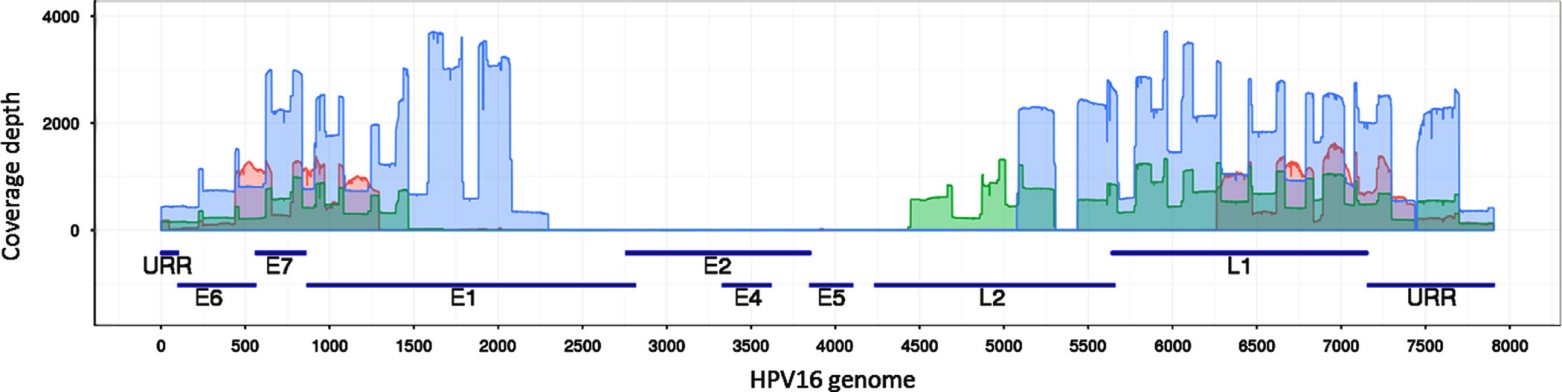
Coverage depth by nucleotide position for three of the 26 HPV16 samples exhibiting large central deletions. Three samples with a large central deletion were randomly chosen to illustrate the coverage depth pattern. The three sequence coverage plots (shown in blue, green and red) are over-laid upon the ORFs (early genes: E6, E7, E1, E2/E4, E5; late genes: L2, L1) and the upstream regulatory region (URR) and corresponding nucleotide position of the HPV16 genome. (For interpretation of the references to color in this figure legend, the reader is referred to the web version of this article.)

HPV16 whole-genome sequencing and Ion Torrent deep coverage enabled us to identify contiguous amplicons with very low or no sequence reads, interpreted as genome deletions. Twenty-six women contained large central deletions 777 to 4940 bp in size (Fig. 2a, group II). Deleted regions were visualized in the Integrative Genomics Viewer (IGV) [37] and identified based on an abrupt drop in the sequence read depth from >100× to approximately zero. Complete coverage data for the 26 deletions and flanking sequences can be found in Supplemental Fig. 3. The deleted region often included a fragment within the E2 gene, whereas the E6, E7 and URR regions generated high sequence read numbers (Fig. 3). The samples with these deletions were strongly associated with cancer (OR of 27.3, 95% CI 3.3–222, *P*=0.002); occurring in 0.5% of controls, 3.5% of cervical precancers and 13.5% of cancer cases.

The deep coverage, often surpassing 500×, enabled the identification of frequent HPV16 variant lineage co-infections. Co-infections were suspected in women with multiple ‘heterozygous’ allele calls (HPV is a monoploid genome). The co-infections were confirmed by the identification and visualization of multiple lineage-specific sequence variants occurring in shared sequence reads, representing two separate HPV16 variant lineage molecules, using IGV (see example in Fig. 1). The deep read depths allowed estimation of the percent infection with each HPV16 variant lineage with a threshold of 1% for the less abundant variant in an HPV16 mixed infection.

## 4 Discussion

We developed and implemented a high-throughput, NGS technique based on Ampliseq and Ion Torrent technology to investigate the nucleotide changes associated with human papillomavirus oncogenicity. This report focused on the details of the new assay; to demonstrate the importance of the development, we also described selected applications of this technology to study HPV16 genomes in a large set of samples from the first laboratory runs of a large population-based study. To validate this approach we compared the nucleotide sequence and coverage of a subset of samples sequenced in parallel by overlapping PCR and Sanger sequencing of PCR products, a gold standard of HPV genomics. Concordance of nucleotide sequences across the genome was excellent, with >99.9% agreement. Overall, the genome sequences data quality metrics for the 796 samples were very high; the concordance among duplicates run on each plate exceeded 99%, and 86% of samples had a 90% coverage of at least 25× with 96% of samples having at least 80% coverage of 25× or greater.

We first used our pipeline to categorize the HPV16 genomes into well-established variant lineages [12] to test HPV16 sequence variation and cervical cancer risk at the haplotype level. Dichotomizing the HPV16 genomes to the deepest phylogenetic branching, A (European) and B/C/D (non-European) variant lineages, we observed a higher risk of precancer/cancer (CIN3+) for the non-European variant lineages, consistent with our and others previous results [see [12] for review]. This motivates a very large and detailed exploration of finer HPV16 genetic variation among thousands of precancer/cancer cases and controls from the KPNC cohort, which is now possible with our high-throughput assay and is underway. A goal is to use the genetic data to understand the mechanistic basis of specific genomic influences on the natural history of HPV infections and cervical lesions.

Our large sequence-based data enabled us to make several novel observations, previously unachievable with Sanger or targeted sequencing, which deserve separate attention. With this dataset, we are able to define a very large number of HPV16 lineages and SNPs, suggesting that HPV16 actually represents hundreds of distinct viruses. HPV researchers have tended to study HPV16 as a categorical, specific entity. Instead, HPV16 should be thought of as a group of very closely related, genetically-stable viruses, and epidemiologic studies of HPV can now focus on a viral “isolate” level. For example, it is conceivable that using NGS methods, viral transmission studies between sexual networks can be performed with greater precision as to which partner transmitted a particular viral isolate. Longitudinal natural history studies of HPV16 that have observed gaps in HPV positivity (e.g., repeated detection following a period of negative testing) have been performed mainly at the level of individual genotypes. Instead, it will now be possible with greater accuracy to determine whether re-appearing specific HPV16 genetic isolates represents re-emergence from a poorly defined latent state, or acquisition of a new infection.

We were able to annotate numerous novel SNPs and CpG changes across the HPV16 genome that may impact infection outcome. HPV16 CpG site specific methylation has been suggested to be a diagnostic biomarker of risk of cervical precancer/cancer among HPV-positive women [38], [40], [41]. Methylation of CpG sites located in the E2/E4, L2, L1 and URR gene regions have been associated with infection outcome [see [42] for review], and we observed known CpG site losses and CpG site gains in these regions, which should allow further characterization of the biology of these sites and their influence on the fitness and pathogenicity of HPV16.

Another observation is the finding of co-infection with multiple, different HPV16 isolates. Previous studies using smaller regions of the HPV16 genome have estimated the frequency of cervical co-infection with multiple HPV16 variant lineages to be low [43], [44], however our preliminary data (analyses underway) indicate that this low rate may be due to the insensitivity of previous methods of detection. Variant lineage determination has usually been done with Sanger sequencing which is known to be relatively insensitive to the detection of multiple sequences in one sample. NGS provides hundreds of individuals sequence reads from each sample, whereas Sanger sequencing provides a ‘consensus’ of the actual sequence. Sanger sequencing can detect approximately 25% for a minority sequence proportion, but with our new single molecule deep sequencing we can estimate down to 1% co-infection proportions. This is possible by noting consistent lineage specific changes in a specific proportion of reads across the genome required for definitive calling of lower level infections. These data will allow the examination of whether circulation of different HPV16 isolates is independent, or whether they interact in some way, potentially influencing the natural history of HPV16 infections. In microdissection studies of cervical pathology samples, the dogma has become that different HPV types do not co-infect cervical cells [45], [46], although lesions produced by different types can abut each other. It will now be possible to determine co-infections of different isolates of HPV16 at the cellular level.

Also at the molecular level, the 26 large contiguous deletions identified and highly associated with cancer are suggestive of a pattern of HPV integration and expansion of a clonal lesion. HPV DNA can integrate into the host genome, sometimes in precancer (CIN3) and especially in cancers [47], [48]. The deletion patterns observed in our study documented decreased amplification of regions consistent with their loss, particularly in ORFs previously shown to be disrupted in integration (i.e., E2, E4, E5 and regions of E1 and L1), whereas the URR and viral oncogenes (E6 and E7) remained intact. Twenty-five of 26 deletions lacked the E2 gene, a finding consistent with a putative loss of E2-mediated repression of E6 and E7 expression or displacement of the viral polyadenylation signal and dissociation of 3′ signals in the HPV16 early gene transcripts by splicing into cellular sequence [49]. More work is needed to determine whether these deletions are indeed signatures of HPV16 integration and lesions with a high probability of progression.

## 5 Conclusions

We are in the process of extending our NGS method from studies of HPV16 whole-genome sequence variation to DNA derived from formalin-fixed paraffin embedded (FFPE) tissues, closely related types in alpha-9, and eventually all the cancer-associated types, attempting to determine why HPV16 is a uniquely powerful carcinogen. In summary, we have developed a NGS method and sequence analysis pipeline that is adaptable to high-throughput, enabling the practical sequencing of thousands of HPV16-containing specimens from epidemiologically sound, informative populations for the evaluation of the genetic basis of HPV carcinogenicity. These NGS data have several advantages over existing approaches and have already enabled the detection of a large number of novel HPV16 SNPs, stable sublineage clades, CpG site changes, large contiguous deletions suggestive of viral integration, and the sensitive detection of variant lineage coinfections. It is not an exaggeration to state that NGS methods signal a new era in the study of HPV and related cancers.

## Footnotes

Appendix A Supplementary data associated with this article can be found in the online version at doi:10.1016/j.pvr.2015.05.004.
