# Supplementary material for: Deep sequencing of HPV16 genomes: A new high-throughput tool for exploring the carcinogenicity and natural history of HPV16 infection
Source: Papillomavirus Res. 2015 Jun 15;1:3–11. doi: 10.1016/j.pvr.2015.05.004 (PMC4669577; doi:10.1016/j.pvr.2015.05.004)
Supplement: Supplementary file 1 — Supplementary material [file mmc1.pdf]

Supplemental Table 1. Custom overlapping HPV16 degenerate primers for **Ion Torrent** complete genome coverage.

**FOR** primer name	Sequence (5'-3')	**REV** primer name	Sequence (5'-3')
HPV16_1_F	ACAGTTACTGCGACGTGAGG	HPV16_1_R	TGGAATCTTTGCTTTTTGTCC
HPV16_2_F	TCAAAAGCCACTGTGTCCTG	HPV16_2_R	TTCATCCTCCTCCTCTGAGC
HPV16_3_F	TTGCAACCAGAGACAACTGA	HPV16_3_R	TTCTGAGAACAGATGGGGCAC
HPV16_4_F	GCGTACAAAGCACACACGTA	HPV16_4_R	CTGTCATTTTCGTTCTCGTCA
HPV16_5_F	ACGGGATGTAATGGATGGTT	HPV16_5_R	TGTTGTTTTGCTTCCTGTGC
HPV16_6_F	TTTAACACAGGCAGAAACAGAGAC	HPV16_6_R	CGCCCTTCTACCTGTAACATC
HPV16_7_F	GCTGCAAAAAGGAGATTATTTG	HPV16_7_R	ATTGCTGCCTTTGCATTACT
HPV16_8_F	TGCGAAACACCACTTACAAA	HPV16_8_R	CATTCCCCATGAACATGCTA
HPV16_8B_F	TGCCAAACACCACTTACAAA	HPV16_8B_R	CATTCCCCATGAACACGCTA
HPV16_9_F	GGTGTATTGCTGCATTTGGA	HPV16_9_R	TACGCAATTTTGGAGGCTCT
HPV16_10_F	TGTGTGTCTCCAATGTGTATGATG	HPV16_10_R	CCCATTGTACCATCGTGATAATTC
HPV16_11_F	ACACGCCAGAATGGATACAA	HPV16_11_R	CACATTGTTGCACAATCCTTT
HPV16_12_F	TGCACAATTGGCAGACACTA	HPV16_12_R	ACCTGTGTTAGCTGCACCAT
HPV16_13_F	AGATGTGATAGGGTAGATGATGGAG	HPV16_13_R	TTGTCATCTATGTAGTTCCAACAGG
HPV16_14_F	GCAGATGCCAAAATAGGTATG	HPV16_14_R	ACTGGATTTCCGTTTTTGTC
		HPV16_14B_R	ACTGGATTTCCGTTTTCGTC
HPV16_15_F	GATTGGTGGTGTTTACATTTCC	HPV16_15_R	CATTCTAGGCGCATGTGTTT
		HPV16_15B_R	CATTCTAGGCGCATTTGTTT
HPV16_16_F	AAAACGATGGAGACTCTTTGC	HPV16_16_R	AGTTGCAGTTCAATTGCTTGT
HPV16_17_F	GTGCCAACACTGGCTGTATC	HPV16_17_R	TGCATATGTCTCCATCAAACTG
HPV16_18_F	AAACATGGATATACAGTGGAAGTGC	HPV16_18_R	ATTACCTGACCACCCGCATG
HPV16_19_F	TGCAGTTTAAAGATGATGCAGA	HPV16_19_R	CGCTGGATAGTCGTCTGTGT
HPV16_20_F	TCTGTGTTTAGCAGCGACGA	HPV16_20_R	CAGTGAGGATTGGAGCACTG
HPV16_20B_F	TCTGTGTTTAGCAGCAACGA		
HPV16_21_F	ACCCCTGCCACACCAATAAG	HPV16_21_R	TATGTCCTGTCCAATGCCATG
HPV16_22_F	CCATAGTACATTTAAAAGGTGATGC	HPV16_22_R	CGCCAGTAATGTTGTGGATG
HPV16_23_F	TACACTTACATATGATAGTGAATGTCAACG	HPV16_23_R	CGTATGTAGACACAGACAAAAGCAG
HPV16_24_F	GTGTGCTTTTGTGTGTCTGCC	HPV16_24_R	TGTGTCGCATTGTTAAGTGATAAC
HPV16_25_F	GGATAACAGCGGCCTCTGC	HPV16_25_R	AAAGTTGGGTAGCCGATGC
HPV16_26_F	GTTCTGCAAAACGCACAAA	HPV16_26_R	GGGGTCTTACAGGAGCAAGT
HPV16_27_F	GTACAGGCGGACGCACTG	HPV16_27_R	GGGATTATTATGTGTAGTAACAGTAGTAACAG
HPV16_28_F	TCAACTGATACCACACCTGCT	HPV16_28_R	GAGACCCTGGTATGGGTGTG
		HPV16_28B_R	GAGACCCCGGTATGGGTGTG
HPV16_29_F	AGCACAAACCCTAACACAGTAACTAG	HPV16_29_R	TAAAATGTATTATCCACATCTATACCTTC

HPV16_29B_F	AGCACAAATCCTAACACAGTAACTAG		
HPV16_30_F	GACCCTGCTTTTGTAACCACTC	HPV16_30_R	GTACGCCTAGAGGTTAATGCTGG
HPV16_31_F	GCTCCAGATCCTGACTTTTTG	HPV16_31_R	AGTIGGTGAGGCTGCATGGG
		HPV16_31B_R	AGTIGGTGAGGCTGCATGTG
HPV16_32_F	GTAGAATTGGTAATAAACAAACACTACG	HPV16_32_R	GGAAGTAATGAAGGAGTTTGGTCAG
HPV16_33_F	TTCCTGCAAATACAACAATTCC	HPV16_33_R	TACTGGGATAGGAGGCAAGTAGAC
HPV16_34_F	CATGTTACGAAAACGACGTAAA	HPV16_34_R	CCAAACTTATTGGGGTCAGG
HPV16_35_F	CTTGCAGTTGGACATCCCTA	HPV16_35_R	CACACCTAATGGCTGACCAC
HPV16_36_F	GTTTGGGCCTGTGTAGGTG	HPV16_36_R	TTCCCCTATAGGTGGTTTGC
HPV16_37_F	AGCAAATGCAGGTGTGGATA	HPV16_37_R	TCCAGTGGAACTTCACTTTTG
HPV16_38_F	CGGCTTTGGTGCTATGGAC	HPV16_38_R	GCAGTAGACCCAGAGTCTTTAATG
HPV16_39_F	TGGTGAAAATGTACCAGACGA	HPV16_39_R	GATATGGCAGCACATAATGACA
HPV16_40_F	ATGGCATTTGTTGGGGTAAC	HPV16_40_R	ACCAAAATTCCAGTCCTCCA
HPV16_41_F	TGTGCAAAATAACCTTAACTGC	HPV16_41_R	TGCGTCCTAAAGGAAACTGA
HPV16_42_F	GGAGGCACACTAGAAGATACTTATAGG	HPV16_42_R	GAGGTGGTGGGTGTAGCTTTTC
HPV16_43_F	AAGGCCAAACCAAAATTTACA	HPV16_43_R	GCATGACACAATAGTTACACAAGC
HPV16_44_F	GTTTGTATGTGCTTGTATGTGCTTG	HPV16_44_R	CGGTTGAAGCTACAAAATGGC
HPV16_45_F	GCCATTTTGTAGCTTCAACCG	HPV16_45_R	CAAGCCAAAAATATGTGCCTAAC
HPV16_46_F	TAAATTACTATGCGCCAACG	HPV16_46_R	ACTAACCGGTTTCGGTTCAA
HPV16_46B_F	TAAATCACTATGCGCCAACG		
HPV16_47_F	CAAACCGTTTTGGGTTACAC	HPV16_47_R	ATGCATAAATCCCGAAAAGC

Supplemental Table 2. Custom overlapping HPV16 PCR primers for **Sanger** complete genome coverage.

Fragment	Primer name	Start position	Sequence (5'-3')
Fragment 1 outer	16.3Fr1.Fout	878 - 897	CAGGTACCAATGGGGAAGAG
Fragment 1 outer	16.3Fr1.Rout	3894 - 3913	GCACACAAAAGCAAAGCAAA
Fragment 1 inner	16.3Fr1.Fin	901 - 920	ACGGGATGTAATGGATGGTT
Fragment 1 inner	16.3Fr1.Rin	3869 - 3888	CGCCAGTAATGTTGTGGATG
Fragment 2 outer	16.3Fr2.Fout	3573 - 3592	GCTCACACAAAGGACGGATT
Fragment 2 outer	16.3Fr2.Rout	6580 - 6601	ATTATTGTGGCCCTGTGCTC
Fragment 2 inner	16.3Fr2.Fin	3710 - 3729	TGGCATTGGACAGGACATAA
Fragment 2 inner	16.3Fr2.Rin	6533 - 6552	TGGGCATCAGAGGTAACCAT
Fragment 3 outer	16.3Fr3.Fout	5933 - 5952	GTTTGGGCCTGTGTAGGTGT
Fragment 3 outer	16.3Fr3.Rout	1078 - 1097	TSTTTTGCTTCCTGTGCAGT
Fragment 3 inner	16.3Fr3.Fin	6047 - 6066	GCAAATGCAGGTGTGGATAA
Fragment 3 inner	16.3Fr3.Rin	1054 - 1073	AACGCATGTGCTGTCTCTGT

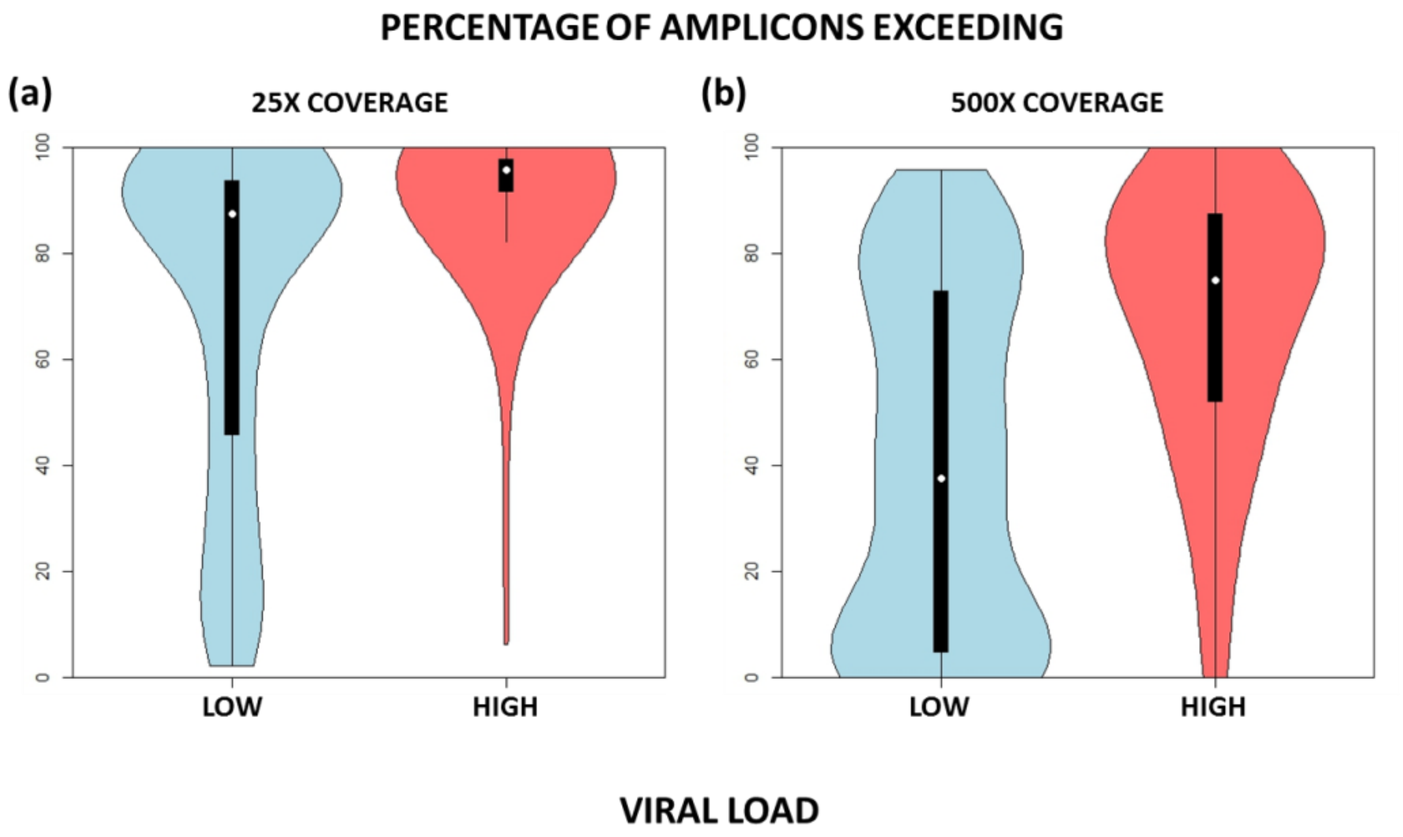

Supplemental Figure 1. Violin plots displaying the relationship between HPV16 viral load (low vs. high) and Ion Torrent sequence coverage greater than **(a)** 25x and **(b)** 500x coverage. Each violin is a box plot embedded into rotated kernel density plots. Medians are illustrated as white dots, black boxes indicate the interquartile range and black lines extend out 1.5 times the interquartile range. Filled regions are fitted density plots that capture the shape of the underlying density mass function.

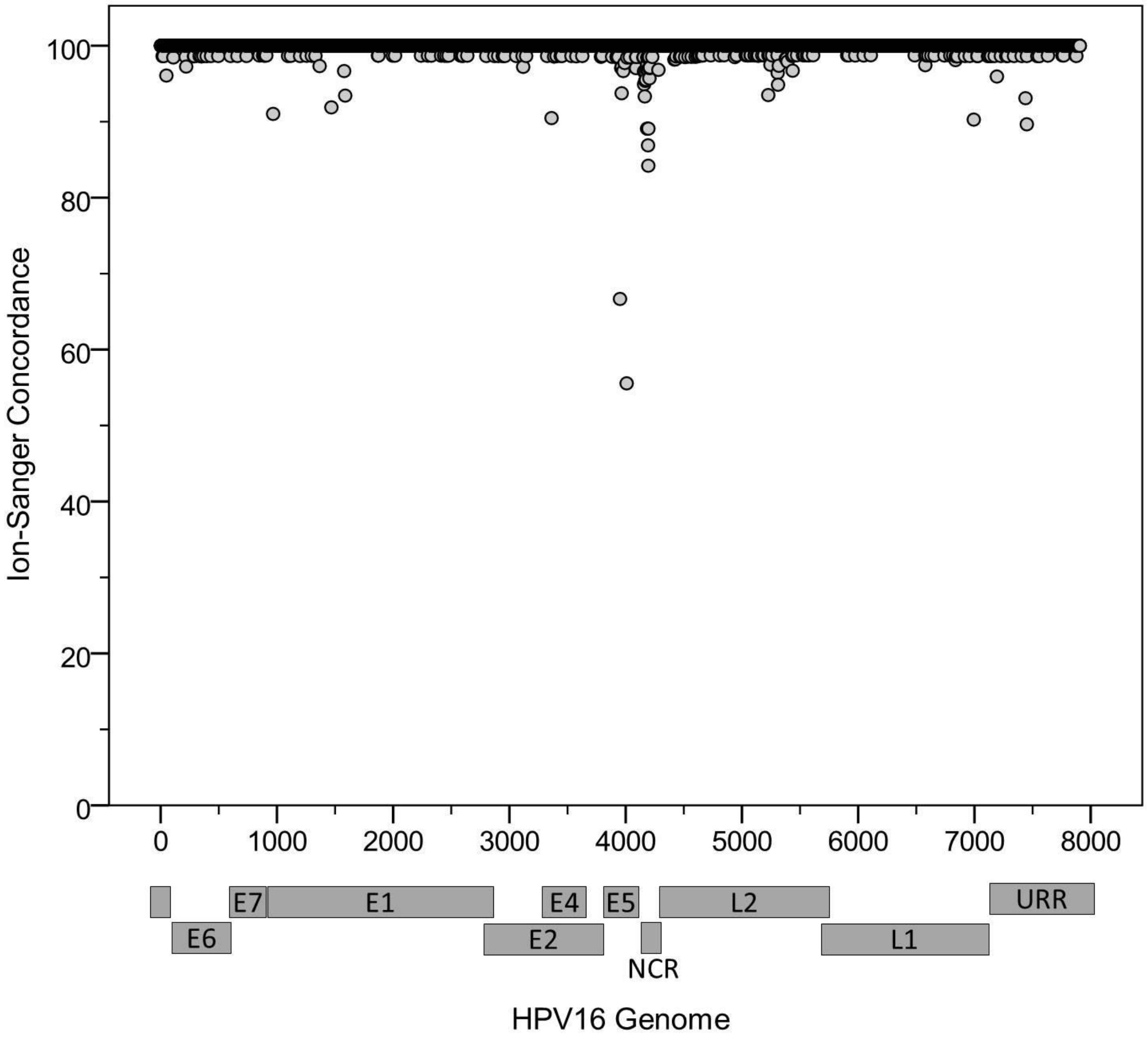

Supplemental Figure 2. Percent Ion Torrent and Sanger sequencing concordance by HPV16 genomic position. The approximate location of the HPV16 genes/regions (E6, E7, E1, E2/E4, non-coding region [NCR], L2, L1, upstream regulatory region [URR]) are illustrated relative to their genomic map position on the x-axis.

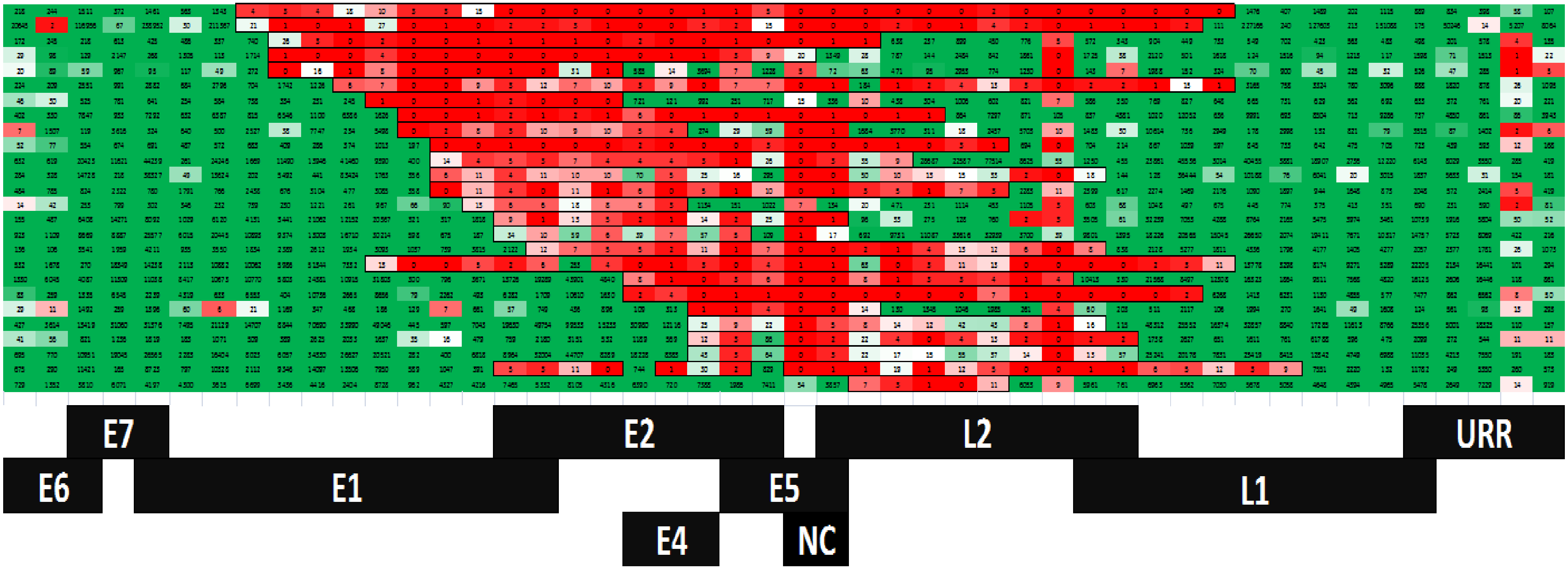

Supplemental Figure 3. Amplicon heat map of 26 HPV16 samples containing large central deletions. The heat map illustrates sequence depth across 48 overlapping amplicons (columns 1-48) for the 26 samples with deletions (rows 1-26), the sequence depths ≥100x are green cells, depths of 15x white cells and depths ≤2x red cells. The physical boundaries of each deletion are defined by the bold outline surrounding all amplicons believed to be deleted in a given sample. Read counts within the deleted regions (red cells) are strikingly low compared to the read counts of flanking amplicons. The exaggerated overlap of adjacent HPV16 genes and regions (early genes: E6, E7, E1, E2, E4, E5; late genes: L2, L1; the upstream regulatory region, URR; non-coding region, NC) in the map reflects the overlapping design of the amplicons.
